# Polymorphisms in dipeptidyl peptidase 4 reduce host cell entry of Middle East respiratory syndrome coronavirus

**DOI:** 10.1080/22221751.2020.1713705

**Published:** 2020-01-21

**Authors:** Hannah Kleine-Weber, Simon Schroeder, Nadine Krüger, Alexander Prokscha, Hassan Y. Naim, Marcel A. Müller, Christian Drosten, Stefan Pöhlmann, Markus Hoffmann

**Affiliations:** a Infection Biology Unit, German Primate Center, Göttingen, Germany; b Faculty of Biology and Psychology, University Göttingen, Göttingen, Germany; c Charité-Universitätsmedizin Berlin, corporate member of Freie Universität Berlin, Humboldt-Universität zu Berlin, and Berlin Institute of Health, Institute of Virology, Berlin, Germany; d Institute of Virology, University of Veterinary Medicine Hannover, Hannover, Germany; e Research Center for Emerging Infections and Zoonoses, University of Veterinary Medicine Hannover, Hannover, Germany; f Department of Physiological Chemistry, University of Veterinary Medicine Hannover, Hannover, Germany; g German Centre for Infection Research, associated partner Charité, Berlin, Germany; h Martsinovsky Institute of Medical Parasitology, Tropical and Vector Borne Diseases, Sechenov University, Moscow, Russia

**Keywords:** Middle East respiratory syndrome coronavirus, spike glycoprotein, dipeptidyl peptidase 4, polymorphisms, receptor binding

## Abstract

Middle East respiratory syndrome (MERS) coronavirus (MERS-CoV) causes a severe respiratory disease in humans. The MERS-CoV spike (S) glycoprotein mediates viral entry into target cells. For this, MERS-CoV S engages the host cell protein dipeptidyl peptidase 4 (DPP4, CD26) and the interface between MERS-CoV S and DPP4 has been resolved on the atomic level. Here, we asked whether naturally-occurring polymorphisms in DPP4, that alter amino acid residues required for MERS-CoV S binding, influence cellular entry of MERS-CoV. By screening of public databases, we identified fourteen such polymorphisms. Introduction of the respective mutations into DPP4 revealed that all except one (Δ346-348) were compatible with robust DPP4 expression. Four polymorphisms (K267E, K267N, A291P and Δ346-348) strongly reduced binding of MERS-CoV S to DPP4 and S protein-driven host cell entry, as determined using soluble S protein and S protein bearing rhabdoviral vectors, respectively. Two polymorphisms (K267E and A291P) were analyzed in the context of authentic MERS-CoV and were found to attenuate viral replication. Collectively, we identified naturally-occurring polymorphisms in DPP4 that negatively impact cellular entry of MERS-CoV and might thus modulate MERS development in infected patients.

## Introduction

Middle East respiratory syndrome coronavirus (MERS-CoV) is an enveloped virus with a single-stranded RNA genome of positive polarity. It belongs to the *Coronaviridae* family (genus *Betacoronavirus*), which is part of the order *Nidovirales*. MERS-CoV was isolated in 2012 from the sputum of a 60 year old man suffering from acute pneumonia and renal failure in Saudi Arabia [1]. Since its discovery, MERS-CoV has caused 2,442 human infections of which 842 (34.5%) had a fatal outcome (as of May, 2019) [2]. Dromedary camels are reservoir hosts of MERS-CoV and display only common cold-like symptoms upon infection but constitute the main source of human infections. Transmission to humans occurs via close contact to animals or contaminated animal products [3–6]. Human-to-human transmissions seem limited and were mainly observed in health care settings, leading to MERS outbreaks in hospitals [7–12]. Finally, differences in the tissue specific expression of the cellular receptor for MERS-CoV, DPP4, were recently suggested to account for the differences in MERS-CoV transmission and disease induction in camels and humans, respectively [13,14].

In order to infect a host (cell) and replicate, MERS-CoV has to deliver its genome into the cellular cytoplasm for gene translation and genome replication. This process is facilitated by the viral spike (S) glycoprotein, a type-I transmembrane protein embedded in the viral envelope. For host cell entry, the surface unit, S1, of MERS-CoV S binds to the cellular type-II transmembrane protein dipeptidyl peptidase 4 (DPP4, CD26) [15]. The structure of the interface between DPP4 and MERS-CoV-S was resolved on the atomic level and fifteen residues in DPP4 were found to make direct contact with residues in the viral S protein [16]. Upon DPP4 engagement, MERS-CoV S undergoes proteolytic activation through the cellular serine protease TMPRSS2 or the endosomal cysteine protease cathepsin L [17–19], which allows the transmembrane unit, S2, of MERS-CoV S to fuse the viral membrane with cellular membranes.

DPP4 is a prolyl oligopeptidase that is expressed in various tissues [20] and involved in multiple biological processes including T-cell activation [21], control of the activity of growth factors, chemokines and bioactive peptides [22–24], and regulation of the glucose metabolism [25]. Mature DPP4 is embedded in the plasma membrane as a homodimer and each monomer consists of an N-terminal cytoplasmic domain, followed by a transmembrane domain and a large ectodomain, which can be further subdivided into a short stalk domain, a glycosylation-rich and a cysteine-rich region as well as the C-terminal catalytic domain (α/β-hydrolase domain) [26]. Polymorphisms in the DPP4 gene were implicated in several diseases and conditions, including diabetes [27,28] and myocardial infarction [29] but their potential impact on MERS-CoV infection has not been analyzed.

We asked whether naturally-occurring amino acid polymorphisms in DPP4 residues making contact with MERS-CoV S have an impact on MERS-CoV entry. We identified fourteen polymorphisms by screening public databases and introduced the respective mutations into a DPP4 expression plasmid. We identified four mutations that reduced MERS-CoV S binding to DPP4 and MERS-CoV S-driven host cell entry without affecting DPP4 expression at the cell surface.

## Materials and methods

### Analysis of total DPP4 expression by SDS-PAGE and immunoblot

293T cells were transfected with expression vectors for WT or mutant DPP4, or empty expression vector (negative control). At 16 h post transfection, the culture medium was replaced and the cells were further incubated for additional 32 h. Then, the cells were washed with PBS and mixed with 2x SDS-sample buffer (0.03 M Tris-HCl, 10% glycerol, 2% SDS, 0.2% bromophenol blue, 1 mM EDTA). Cell lysis was achieved by incubating the samples for 10 min at room temperature followed by incubation at 96 °C for an additional 10 min. The samples were further loaded on polyacrylamide gels and SDS-PAGE (sodium dodecyl sulfate-polyacrylamide gel electrophoresis) was performed. Next, the proteins were transferred onto nitrocellulose membranes (Hartenstein GmbH) by immunoblotting. The membranes were further blocked by incubation in PBS-T (PBS containing 0.5% Tween 20 and 5% skim milk powder) for 30 min at room temperature. Afterwards, the membranes were incubated overnight at 4 °C with undiluted supernatant of a hybridoma cell line secreting anti-cMYC antibody 9E10 (for DPP4 detection) or PBS-T containing anti-ß-actin (ACTB) antibody (mouse, 1:1,000, Sigma Aldrich). Following three washing intervals with PBS-T, the membranes were further incubated with PBS-T containing horseradish peroxidase-conjugated anti-mouse antibody (goat, 1:5,000, Dianova) for 1 h at room temperature before an in house-prepared enhanced chemiluminescent solution (0.1 M Tris-HCl [pH 8.6], 250 µg/ml luminol, 1 mg/ml para-hydroxycoumaric acid, 0.3% H_2_O_2_) was added and signals were recorded using the ChemoCam imaging system and the ChemoStar Professional software (Intas Science Imaging Instruments GmbH).

In order to quantify the signal intensity of the protein bands, the program ImageJ (FIJI distribution) [30] was used. To account for differences in the total protein content of the samples and variations, we normalized the DPP4 signals against the respective signals of the loading control (ACTB).

### Cell culture

293T (human kidney cells, DSMZ no. ACC 635), BHK-21 (hamster kidney cells, DSMZ no. ACC 61) and Vero 76 (African green monkey kidney cells, kindly provided by Andrea Maisner, Philipps-University Marburg) were cultivated in Dulbecco’s modified Eagle medium (PAN-Biotech) while Caco-2 cells (human colorectal adenocarcinoma cells) were cultivated in Minimum Essential Medium (ThermoFisher Scientific). The media were supplemented with 10% fetal bovine serum (Biochrom), 100 U/ml of penicillin and 0.1 mg/ml of streptomycin (PAN-Biotech). All cell lines were incubated at 37 °C and 5% CO2 in a humidified atmosphere. For subcultivation and seeding, cells were washed with phosphate-buffered saline (PBS) and detached by incubation with trypsin/EDTA solution (PAN-Biotech) (BHK-21, Vero 76 and Caco-2) or by resuspending the cells in culture medium (293T). Transfection of 293T and BHK-21 cells was carried out by calcium-phosphate precipitation or with the help of ICAFectin-441 (In-Cell-Art) or FuGENE HD (Promega).

### Plasmids and generation of DPP4 mutants

All DPP4 mutants were generated based on a pcDNA3.1/Zeo(+)-based expression vector in which the coding sequence for human DPP4 (GenBank: XM_005246371.3) containing an C-terminal cMYC epitope was inserted into via BamHI/EcoRI restriction sites. The following aa (amino acid) substitutions were introduced via overlap-extension PCR: K267E, K267N, Q286K, T288I, T288S, A289V, A291P, A291V, R317K, Y322H, I346T, I346V and K392N. In addition, a deletion mutant was generated that lacks aa residues 346–348 (Δ346–348). Information on DPP4 polymorphisms was retrieved from the Ensembl database (https://www.ensembl.org/index.html) [31] and the Single Nucleotide Polymorphism Database (dbSNP) of the National Center for Biotechnology Information (NCBI) (https://www.ncbi.nlm.nih.gov/snp) [32], and is based on data provided by the gnomAD database (Genome Aggregation database, https://gnomad.broadinstitute.org/), TOPMed program (Trans-Omics for Precision Medicine, https://www.nhlbiwgs.org/), ExAC consortium (Exome Aggregation Consortium, http://exac.broadinstitute.org/) [33] and the 1000G project (1,000 genomes project, http://www.internationalgenome.org/) [34] (For detailed information see Supplementary Table 1).

We further utilized pCAGGS-based expression vectors for vesicular stomatitis virus (VSV) glycoprotein (G), MERS-CoV S wildtype (WT) and MERS-CoV S (D510G) (the latter two either untagged or equipped with a C-terminal V5 epitope) that have been described elsewhere [35–37]. In addition, a previously described expression vector for angiotensin converting enzyme 2 was employed [38]. Similar to the strategy used for the generation of DPP4 mutants, we employed the overlap-extension PCR technique to introduce a single mutation into the MERS-CoV S open reading frame, thus generating untagged and V5-tagged MERS-CoV S (D539N).

Soluble S comprising the S1 subdomain of MERS-CoV S (aa residues: 1–747) fused to a human IgG Fc tag was generated by inserting the PCR-amplified S1 sequences into the pCG1Fc vector [39] (kindly provided by Georg Herrler, University of Veterinary Medicine Hannover) making use of the BamHI/SalI restriction sites. In addition, we generated an expression vector for the enhanced green fluorescent protein (eGFP) by inserting the eGFP coding sequence, which was PCR-amplified from the pEGFP-C1 vector (Clontech), into the pCAGGS plasmid using the EcoRI/XhoI restriction sites.

All PCR-amplified sequences were subjected to automated sequence analysis (Microsynth SeqLab) to verify their integrity. Sequences of primers used for cloning of the different constructs are available upon request.

### Analysis of DPP4 surface expression by immunofluorescence analysis

BHK-21 cells were grown on coverslips and transfected with the different DPP4 constructs or empty expression vector using ICAFectin-441 (In-Cell-Art) at 24 h post seeding according to the manufacturer’s instructions. After changing the culture medium at 4 h post transfection, the cells were incubated for additional 20 h. Then, the culture medium was aspirated and the cells were washed with PBS, before they were fixed by incubated with PBS containing 4% paraformaldehyde (PBS/PFA) for 15 min at room temperature. Subsequently, the cells were washed with 0.1 M glycine/PBS solution followed by a washing step with PBS. Next, the coverslips were incubated with anti-DPP4 antibody (mouse, diluted 1:200 in PBS containing 1% bovine serum albumin [PBS/BSA], Abcam) for 1 h at 4 °C. For this, the coverslip was put on a drop (20 µl) of antibody solution that was added on a sheet of parafilm inside a humidity chamber (a glass dish in which the parafilm was placed on wet paper tissue). Thereafter, the cells were washed 3x with PBS before incubation with AlexaFluor568-conjugated anti-mouse antibody (goat, 1:1000, diluted in PBS/BSA, ThermoFisher Scientific) for 30 min at 4 °C was performed. Subsequently, the cells were washed 3x with PBS. Finally, the cells were incubated with DAPI (4’,6-diamidino-2-phenylindole, Carl Roth) and mounted in ProLong Gold Antifade Mountant (ThermoFisher Scientific) before they were analyzed using a Zeiss LSM800 (Zeiss) confocal laser scanning microscope and the ZEN imaging software (Zeiss).

### Analysis of DPP4 surface expression by flow cytometry

BHK-21 cells were transfected with expression vectors for WT or mutant DPP4, or empty expression vector (negative control). At 16 h post transfection, the culture medium was replaced and the cells were further incubated for additional 32 h. Then, the cells were washed with PBS, resuspended in PBS/BSA and pelleted by centrifugation (600 x g, 5 min, 4 °C). After aspiration of the supernatant, the cells were resuspended in PBS/BSA containing anti-DPP4 antibody (mouse, diluted 1:100, Abcam) and incubated for 1 h at 4 °C. Next, the cells were pelleted, washed with PBS/BSA, pelleted again, resuspended in PBS/BSA containing AlexaFluor488-conjugated anti-mouse antibody (donkey, diluted 1:500, ThermoFisher Scientific) and incubated for 1 h at 4 °C. Subsequently, the cells were washed (as described above) and resuspended in PBS/PFA for 2 h at 4 °C for fixation. Finally, the cells were washed (as described above) and resuspended in PBS/BSA for flow cytometric analysis using an LSR II flow cytometer and the FACS Diva software (both BD Biosciences). Additional data analysis was carried out using the FCS Express 4 Flow research software (De Novo software). For quantification of DPP4 surface expression, the mean fluorescence intensity (MFI) value of the negative control was subtracted from all samples. For normalization of DPP4 surface expression, values obtained for cells expressing DPP4 WT were set as 100% and the relative surface expression of the respective DPP4 mutants was calculated accordingly.

### Production of soluble MERS-CoV S and binding studies

In order to generate soluble MERS-CoV S for binding studies, 293T cells were transfected with an expression vector for the S1 subunit of MERS-CoV S fused to the Fc fragment of human immunoglobulin G (solMERS-S1-Fc). At 24 h post transfection, the culture medium was exchanged and the cells were further incubated for 24 h before culture supernatants were harvested and freed from cellular debris by centrifugation (4,700 x g, 10 min, 4 °C). The clarified supernatants were loaded on Vivaspin protein concentrator columns with a molecular weight cut-off of 30 kDa (Sartorius) and centrifuged at 4,700 x g at 4 °C until the sample was 10-fold concentrated.

### Analysis of MERS-CoV S / DPP4 interaction with soluble MERS-CoV s1 by flow cytometry

For the binding studies with solMERS-S1-Fc, a similar protocol was followed as described for the analysis of DPP4 surface expression with the exceptions that solMERS-S1-Fc was used instead of the primary antibody (1:10 dilution in PBS/BSA) and that an AlexaFluor488-conjugated anti-human antibody (goat, 1:500 dilution in PBS/BSA, ThermoFisher Scientific) was employed as the secondary antibody. BHK-21 cells transfected with expression vectors for WT or mutant DPP4, ACE2 or empty expression vector (both negative controls) were analyzed by flow cytometry for solMERS-S1-Fc binding using an LSR II flow cytometer and the FACS Diva software (both BD Biosciences). Additional data analysis was carried out using the FCS Express 4 Flow research software (De Novo software). For quantification of solMERS-S1-Fc binding, the MFI value obtained for cells transfected with empty expression vector was subtracted from all samples. Further, binding of solMERS-S1-Fc to cells expressing DPP4 WT was set as 100% and the relative binding efficiencies to cells expressing the respective DPP4 mutants or ACE2 were calculated accordingly.

### Analysis of MERS-CoV S / DPP4 interaction by co-immunoprecipitation

293T cells (grown in 6-well plates) were cotransfected with expression plasmids coding for solMERS-S1-Fc and WT or mutant DPP4. Cells transfected with empty expression vector instead of DPP4 or solMERS-S1-Fc (or both) served as controls. At 48 h posttransfection, cells were washed with PBS and lysed with 500 µl/well NP40 lysis buffer (50 mM, Tris-HCl [pH 8.0], 150 mM NaCl, 1.0% [v/v] NP-40, 1 tablet/100 ml of *Complete* protease inhibitor cocktail [Roche]) by incubation for 45 min on ice. Lysates were centrifuged for 30 min at 16,400 x g at 4 °C, before 400 µl of the supernatant were mixed with 50 µl of protein A-sepharose (1 g protein A-sepharose [Sigma-Aldrich] in 4 ml PBS) while the residual 100 µl of the cell lysate were mixed with 100 µl 2x SDS-sample buffer and incubated for 15 min at 96 °C (These samples were later analyzed to confirm comparable total protein levels [via detection of ACTB] as well as comparable DPP4 and solMERS-S1-Fc levels before the co-immunoprecipitation [co-IP] step.). Following incubation of the lysate/protein A-sepharose mixtures for 2 h at 4 °C in an overhead shaker, the samples were centrifuged for 5 min at 16,400 x g at 4 °C to pellet the protein A-sepharose/solMERS-S1-Fc/DPP4-complexes. After aspiration of the supernatant, 500 µl of NP40 lysis buffer (without protease inhibitors) were added and the cells were mixed by vortexing, before being centrifuged again. This washing routine was repeated three times, before finally 50 µl of 2x SDS-sample buffer were added to the pelleted complexes and the samples were further incubated for 15 min at 96 °C. Thereafter, the samples were subjected to SDS-PAGE and Western blot analysis (see above). Detection of DPP4 (lysate and co-IP samples) and ACTB (lysate samples) was carried out as described above. solMERS-S1-Fc was detected (lysate and co-IP samples) by incubation with a peroxidase-conjugated anti-human antibody (goat, 1:5,000, Dianova).

Signal intensities of the protein bands were quantified as described above. Further, signals obtained for DPP4 were normalized against the respective signals for solMERS-S1-Fc in order to account for variations in transfection efficiency and sample processing.

### Analysis of MERS-CoV S / DPP4 interaction using soluble DPP4 Ligand

For the binding studies with soluble DPP4, a similar protocol was followed as described for the analysis of binding of solMERS-S1-Fc with the exceptions that a soluble DPP4 fused to the Fc region of human IgG (solDPP4-Fc, Acro Biosystems) was used instead of solMERS-S1-Fc (1:200 dilution in PBS/BSA) and that an AlexaFluor488-conjugated anti-human antibody (goat, 1:500 dilution in PBS/BSA, ThermoFisher Scientific) was employed as the secondary antibody. 293T cells transfected with expression vectors for WT or mutant (D510G and D539N) MERS-CoV S, or empty expression vector (negative control) were analyzed by flow cytometry for solDPP4-Fc binding using an LSR II flow cytometer and the FACS Diva software (both BD Biosciences). Additional data analysis was carried out using the FCS Express 4 Flow research software (De Novo software). For quantification of solDPP4-Fc binding, the MFI value obtained for cells transfected with empty expression vector was subtracted from all samples. Further, binding of solDPP4-Fc to cells expressing MERS-CoV S WT was set as 100% and the relative binding efficiencies to cells expressing the respective MERS-CoV S mutants were calculated accordingly.

### Generation of rhabdoviral pseudotypes and transduction studies

We employed a previously described protocol for the generation of VSV pseudotype particles (VSVpp) that is based on a replication-deficient VSV vector that lacks the genetic information for VSV-G but instead contains the genetic information for eGFP and firefly luciferase (fLuc) as reporters of transduction efficiency (VSV*ΔG-fLuc, kindly provided by Gert Zimmer, Institute of Virology and Immunology, Mittelhäusern/Switzerland) [37,40]. In brief, 293T cells transfected with expression vectors for MERS-CoV S, VSV-G (positive control) or empty expression vector (negative control) were inoculated with VSV*ΔG-fLuc for 1 h before being washed with PBS and further incubated for 16 h with culture medium that was supplemented with anti-VSV-G antibody (I1, mouse hybridoma supernatant from CRL-2700; ATCC) (except for cells expressing VSV-G). The produced VSVpp were inoculated onto BHK-21 cells expressing WT or mutant DPP4, or no DPP4 (empty expression vector, negative control) and incubated for 16–18 h before fLuc activity in cell lysates was quantified as an indicator for transduction efficiency using the Beetle-Juice kit (PJK) and a plate luminometer (Hidex) [41].

### MERS-CoV infection and quantification of viral titers

BHK-21 cells were transfected with expression vectors for wildtype or mutant DPP4 (K267E or A291P), or empty expression vector (negative control) using FuGENE HD (Promega) according to the manufacturer’s instructions. At 24 h posttransfection, the cells were infected with MERS-CoV (Human betacoronavirus 2c EMC/2012, MERS-CoV EMC-2012, GenBank accession number: JX869059) at a multiplicity of infection of 0.01 for 1 h. Thereafter, the inoculum was removed and the cells were washed 3x with PBS before fresh medium was added and the first sample (time point 0 h postinfection) was taken. The cells were further incubated and additional samples were taken at 24 and 48 h postinfection. Viral titers in the culture supernatant were analyzed by quantitative reverse-transcriptase PCR, using the upE assay according to a published protocol [42]. In brief, viral RNA was isolated from cell culture supernatant using the NucleoSpin RNA Virus kit (Macherey-Nagel), reverse-transcribed into cDNA using the Superscript III one step RT–PCR system (ThermoFisher Scientific) and analyzed on a LightCycler 480 qPCR cycler platform (Roche) with primers and conditions as specified for the upE assay [42]. In vitro-transcribed standard samples containing defined amounts of MERS-CoV fragments (10, 100, 1,000 and 10,000 copies) were included for absolute quantification as genome equivalents (GE).

### Protein structure visualization

The DPP4 protein structure (4PV7) [43] and the structure of the complex formed by the MERS-CoV S receptor binding domain bound to DPP4 (4L72) [16] were retrieved from the Research Collaboratory for Structural Bioinformatics Protein Database (RSCB PDB, https://www.rcsb.org/). Structure visualization and colorization was performed using the YASARA software (http://www.yasara.org/index.html) [44] and UCSF Chimera version 1.14 (developed by the Resource for Biocomputing, Visualization, and Informatics at the University of California, San Francisco) [45].

### Statistical analysis

One-way or two-way analysis of variance (ANOVA) with Dunnett’s posttest was used to test for statistical significance. Only *p* values of 0.05 or lower were considered statistically significant (*p* > 0.05 [ns, not significant], *p* ≤ 0.05 [*], *p* ≤ 0.01 [**], *p* ≤ 0.001 [***]). For all statistical analyses, the GraphPad Prism 7 software package was used (GraphPad Software).

## Results

### Identification of polymorphisms in DPP4 that alter amino acid residues which make contact with MERS-CoV S

**Figure 1.**
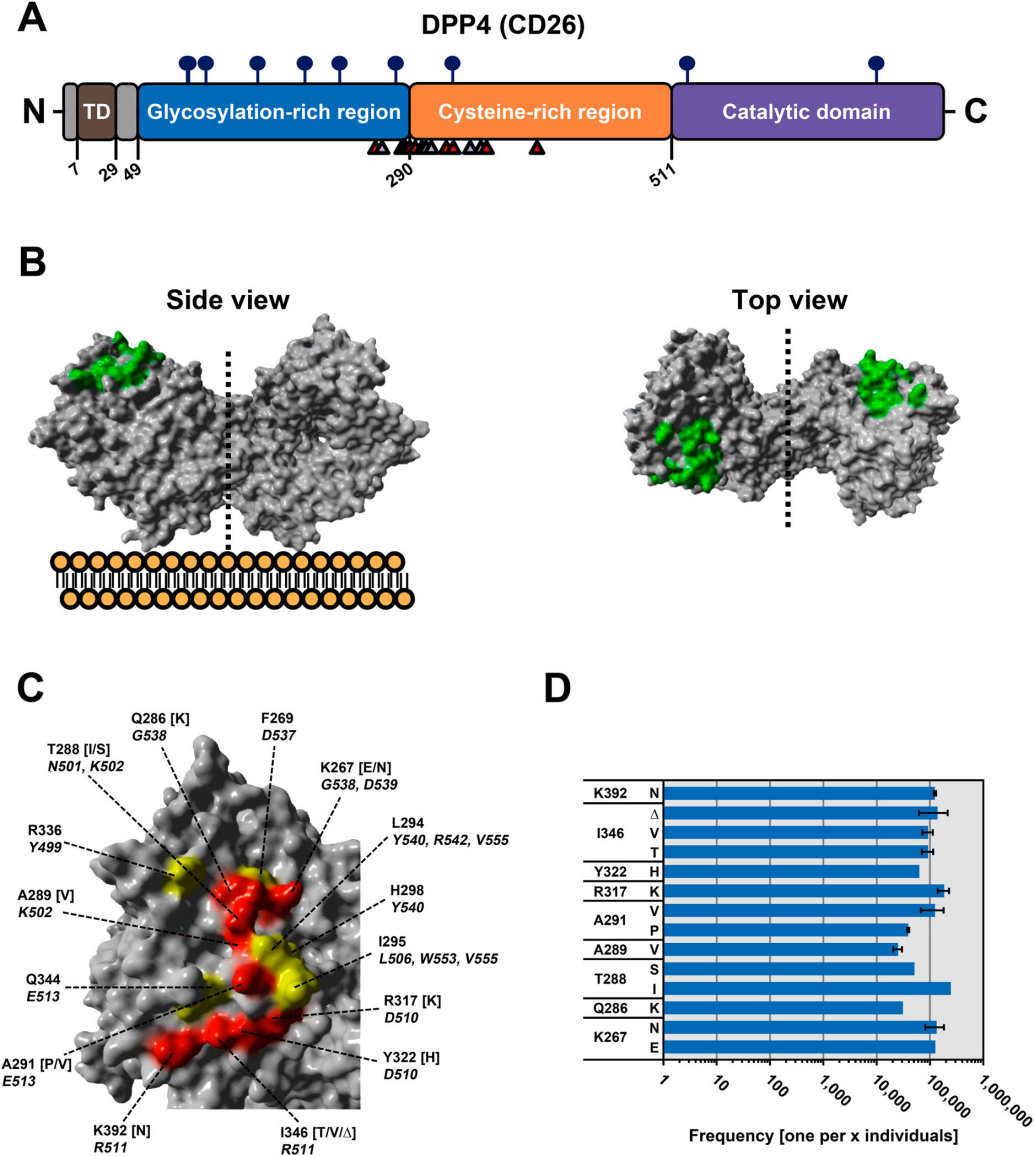
Identification of polymorphic amino acid residues in DPP4 at the binding interface with MERS-CoV S. (A) Schematic representation of DPP4 (CD26). Highlighted are the transmembrane domain (TD, brown), glycosylation-rich (blue) and cysteine-rich (orange) regions, and the catalytic domain (purple). Circles with sticks represent glycosylation sites, while small numbers indicate the amino acid residues. Triangles below the domains highlight the positions of amino acid residues that directly interact with MERS-CoV S (grey triangles mark residues for which no polymorphism has been reported, while red triangles indicate polymorphic residues). (B) Side (left) and top (right) view of homodimeric DPP4 (the dotted line indicates the border between the two monomers and the cellular plasma membrane is schematically depicted below the side view model of DPP4). The protein model was constructed on the published crystal structure (4PV7) deposited in RSCB PDB and the binding interface with MERS-CoV S has been highlighted (green). (C) Close-up on the DPP4 residues that directly interact with MERS-CoV S and for which no polymorphic (yellow) or polymorphic (red) residues have been reported. In addition, the specific residues in DPP4 (regular letters and numbers), including the respective polymorphic residues (letters in brackets), and the corresponding interacting residues in MERS-CoV S (italicized letters and numbers) are indicated. (D) Frequency of polymorphic DPP4 residues in the human population. Public databases (see Supplementary Table 1 and the materials and methods section for detailed information) were screened for the frequency of the polymorphic residues under study (y-axis). Error bars indicate standard error of the mean (SEM) and refer to polymorphic residues found in more than one database.

The binding interface between MERS-CoV S and the cellular receptor DPP4 was resolved by Wang and colleagues using crystallography, revealing the interacting amino acid residues for each binding partner [16]: Fourteen residues of MERS-CoV S (Y499, N501, K502, L506, D510, R511, E513, D537 G538, D539, Y540, R542, W553 and V555) interact with a total of fifteen residues in DPP4 (K267, F269, Q286, T288, A289, A291, L294, I295, H298, R317, Y322, R336, Q344, I346 and K392) [16], which are distributed over the glycosylation-rich domain and the cysteine-rich domain (Figure 1A-C).

In order to identify polymorphic residues in DPP4 that contact MERS-CoV S, we screened public databases that provide information on polymorphic amino acid residues based on data derived from different bio projects (i.e. gnomAD, TOPMed, ExAC, 1000G; more information is given in the Materials and Methods section). By this method we found that nine out of the fifteen DPP4 residues interacting with MERS-CoV S are polymorphic (K267, Q286, T288, A289, A291, R317, Y322, I346 and K392) (Figure 1C). While five of these residues can be replaced by only a single different amino acid residue (Q286[K], A289[V], R317[K], Y322[H] and K392[N]) the remaining four residues can be replaced by two different amino acid residues (K267[E/N], T288[I/S], A291[P/V] and I346[T/V]) or can even be absent from DPP4 (I346Δ) (Figure 1C-D and Supplementary Table 1). Finally, the frequency of these polymorphisms in the human population is low, ranging roughly from 1:19,000 (A289V) to 1:245,000 (T288I) (Figure 1D and Supplementary Table 1).

### DPP4 polymorphisms are compatible with robust DPP4 expression and localization at the cell surface

**Figure 2.**
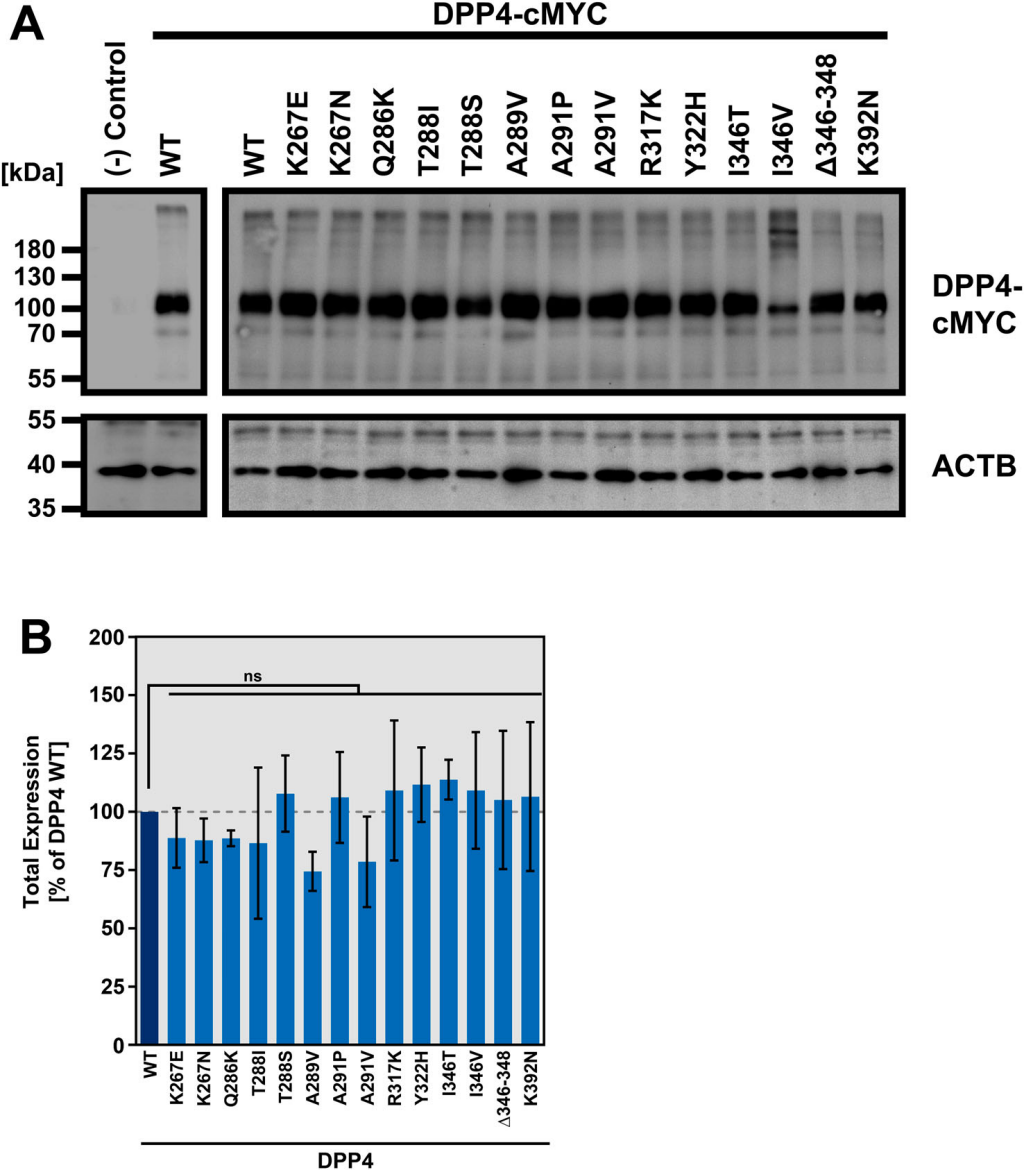
DPP4 harboring polymorphic amino acid residues at the binding interface with MERS-CoV S are robustly expressed. (A) Wildtype (WT) and mutant DPP4 were expressed in 293T cells (cells transfected with empty expression vector served as negative control). Whole cell lysates (WCL) were prepared and analyzed for DPP4 expression by SDS-PAGE under non-reducing conditions and WB using a primary antibody targeting the C-terminal cMYC epitope and a peroxidase-conjugated secondary antibody. Further, expression of beta-actin (ACTB) was analyzed as a loading control. Shown are the expression data from a representative experiment. Numbers at the left indicate the molecular weight in kilodalton (kDa). (B) Quantification of total DPP4 expression in WCL. After normalization of DPP4 band intensities with that of the corresponding ACTB bands. DPP4 WT expression was set as 100% and the relative expression of mutant DPP4 was calculated accordingly. Presented are the combined data of three independent experiments with error bars indicating the SEM. No statistical significance for differences in total expression between WT and mutant DPP4 was observed by one-way analysis of variance with Dunnett’s posttest (*p* > 0.05, not significant [ns]).

**Figure 3.**
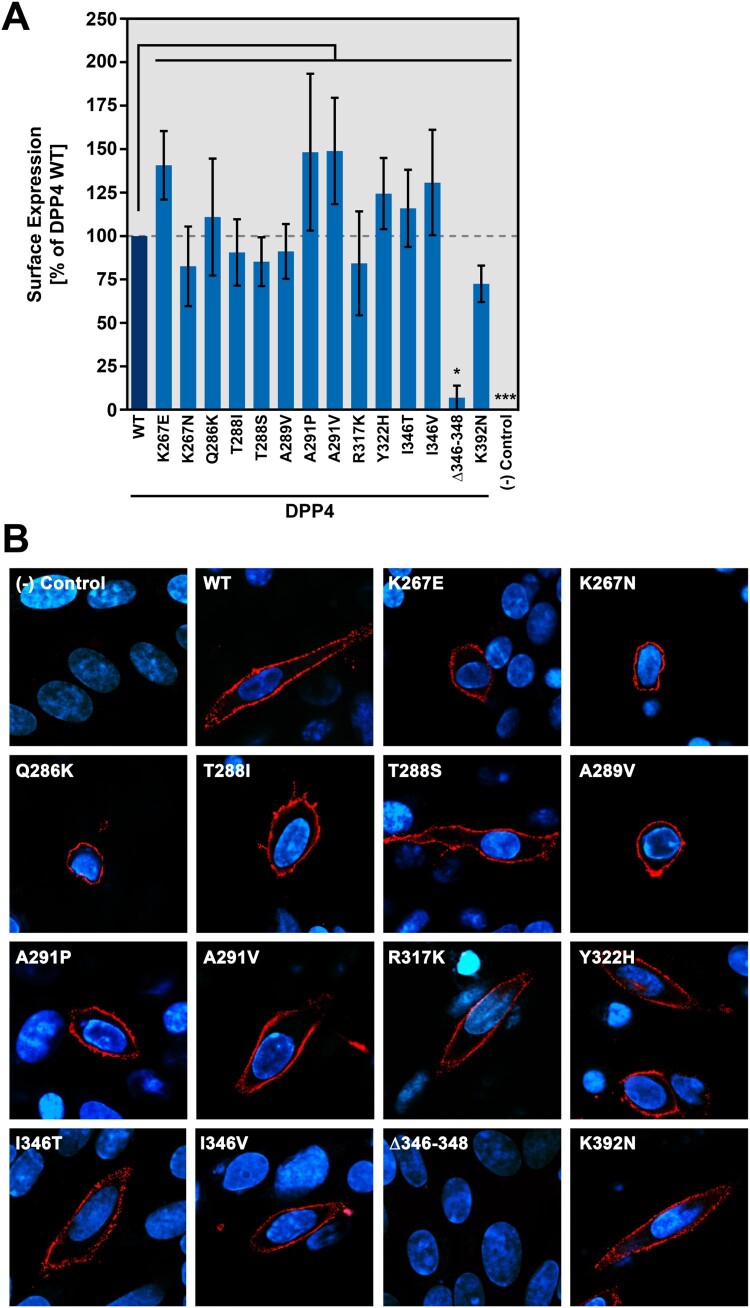
DPP4 harboring polymorphic amino acid residues at the binding interface with MERS-CoV S are efficiently transported to the cell surface. (A) Wildtype (WT) and mutant DPP4 were expressed in BHK-21 cells (cells transfected with empty expression vector served as negative control). Surface expressed DPP4 was stained by subsequent incubation of the non-permeabilized cells with a primary antibody that targets the DPP4 ectodomain and an AlexaFluor488-conjugated secondary antibody. Fluorescent signals representing surface-expressed DPP4 were analyzed by flow cytometry and the mean fluorescence intensity (MFI) values for each sample were calculated. For normalization, the MFI value of the negative control was subtracted from all samples. Further, surface expression of DPP4 WT was set as 100% and the relative surface expression of the DPP4 mutants was calculated accordingly. Shown are the combined data of three experiments with error bars indicating the SEM. Statistical significance for differences in surface expression between WT and mutant DPP4 was tested by one-way analysis of variance with Dunnett’s posttest (*p* > 0.05, not significant; *p* ≤ 0.05, *). (B) DPP4 surface expression was further analyzed by immunofluorescence analysis. For this, DPP4 WT or DPP4 mutants were expressed in BHK-21 cells grown on coverslips (cells transfected with empty expression vector served as negative control). After fixation of the cells, surface expressed DPP4 was stained by subsequent incubation of non-permeabilized cells with a primary antibody that targets the DPP4 ectodomain and an AlexaFluor568-conjugated secondary antibody. In addition, cellular nuclei were stained with DAPI. Finally, images were taken using a confocal laser scanning microscope at a magnification of 80x.

We next introduced the polymorphisms in a DPP4 expression plasmid. Western blot analysis and signal quantification revealed that all resulting DPP4 variants were robustly expressed and total expression levels were comparable (Figure 2A-B). As DPP4 needs to be transported to the plasma membrane to be engaged by MERS-CoV S for host cell entry, we next investigated whether the presence of the polymorphic DPP4 residues has an impact on DPP4 cell surface localization. For this, we performed flow cytometry and confocal laser scanning microscopy, using transfected BHK-21 cells and an antibody targeting the DPP4 ectodomain. We found that all DPP4 variants but one, a deletion variant lacking amino acid residues 346–348 (Δ346–348), displayed comparable cell surface expression levels (Figure 3A-B).

### Polymorphisms at positions 267 and 291 in DPP4 reduce S protein-driven host cell entry and replication of authentic MERS-CoV

**Figure 4.**
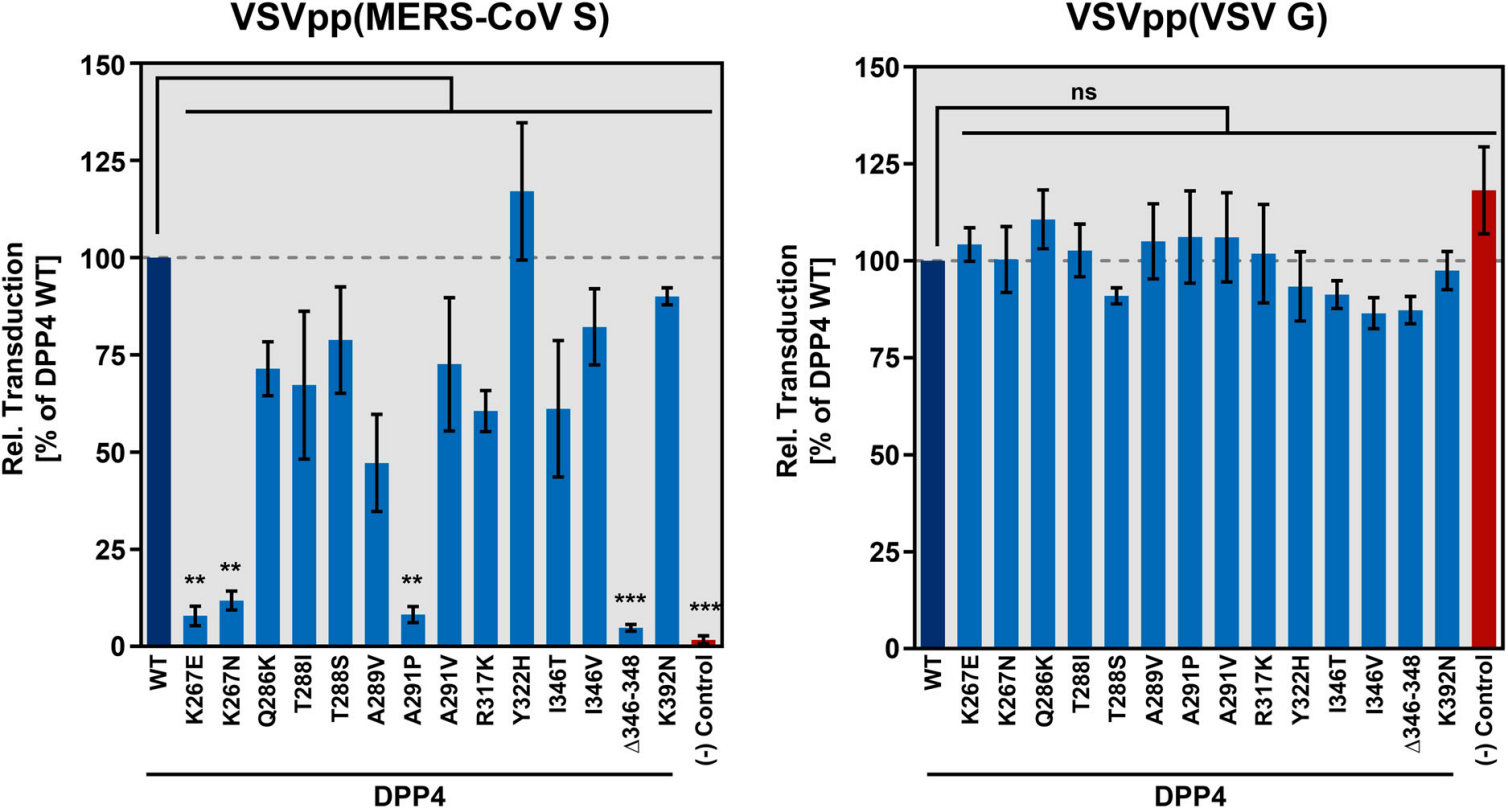
Identification of polymorphic amino acid residues in DPP4 that do not support efficient MERS-CoV S-driven host cell entry. (A) To investigate whether mutant DPP4 support host cell entry driven by MERS-CoV S, we produced vesicular stomatitis virus pseudotype particles (VSVpp) harboring MERS-CoV S (left) or VSV G (control, right). VSVpp were further inoculated on BHK-21 cells expressing wildtype (WT) or mutant DPP4, or cells that were transfected with empty expression vector. At 16 h posttransduction, transduction efficiency was analyzed by measuring the activity of virus-encoded firefly luciferase. Shown are the combined data from three independent experiments (each performed in quadruplicates) for which transduction efficiency of cells expressing DPP4 WT was set as 100%. Error bars indicate the SEM. Statistical significance of differences in transduction efficiency of cells expressing WT or mutant DPP4 was analyzed by one-way analysis of variance with Dunnett’s posttest (*p* > 0.05, not significant [ns]; *p* ≤ 0.05, *; *p* ≤ 0.01, **; *p* ≤ 0.001, ***).

**Figure 5.**
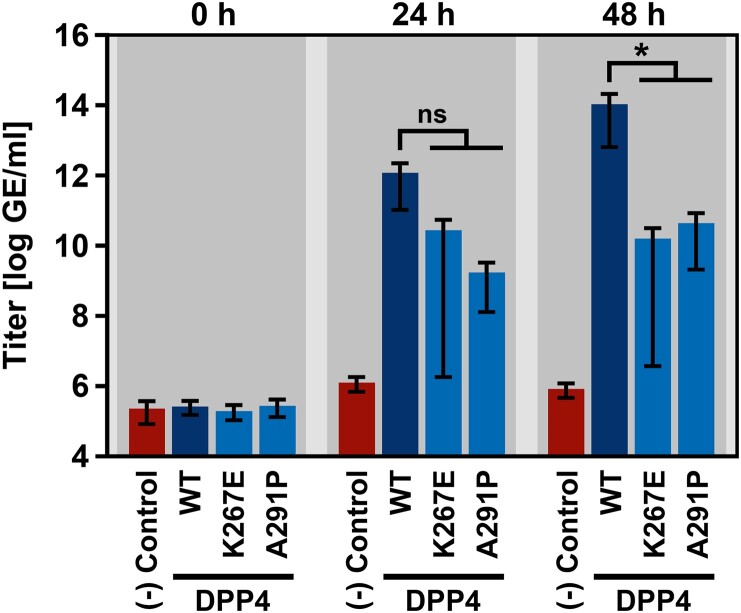
DPP4 harboring polymorphic amino acid residues at the binding interface with MERS-CoV S poorly support replication of live MERS-CoV. Two DPP4 mutants that showed reduced compatibility for MERS-CoV S-driven host cell entry (K267E and A291P) were analyzed in the context of infection and replication of authentic MERS-CoV. For this, BHK-21 cells expressing wildtype (WT) or mutant DPP4, or no DPP4 at all (negative control) were inoculated with MERS-CoV. At 1 h postinfection, the inoculum was removed and the cells were washed before they received fresh culture medium and were further incubated. MERS-CoV replication was analyzed at 0, 24 and 48 h postinfection by determining MERS-CoV genome equivalents (GE) in the culture supernatant (given as GE/ml) by quantitative reverse-transcriptase PCR. Shown are the combined results of three independent experiments (each performed in triplicates). Error bars indicate the SEM. Statistical significance of differences in MERS-CoV replication in cells expressing WT or mutant DPP4 was analyzed by two-way analysis of variance with Dunnett’s posttest (*p* > 0.05, ns; *p* ≤ 0.05, *).

We next investigated whether polymorphic DPP4 residues impact MERS-CoV host cell entry. For this, we made use of vesicular stomatitis virus (VSV) pseudotypes (VSVpp) bearing MERS-CoV S or VSV G, which does not bind to DPP4 and served as negative control [46]. As expected, VSVpp harboring VSV G were able to efficiently transduce BHK-21 target cells irrespective of DPP4 expression. In contrast, transduction of BHK-21 cells mediated by MERS-CoV S critically depended on ectopic expression of human DPP4, in accordance with published findings [47] (Figure 4). Notably, four DPP4 polymorphisms - K267E, K267N, A291P and Δ346–348 - severely reduced MERS-CoV S-driven transduction compared to DPP4 WT (Figure 4). In order to analyze whether the reduction in MERS-CoV S-driven host cell entry would translate into attenuated MERS-CoV replication, we next investigated two DPP4 polymorphisms (K267E and A291P) in the context of infection with authentic MERS-CoV. When followed over a period of two days post infection it was observed that MERS-CoV replication in BHK-21 cells expressing human DPP4 was significantly reduced when DPP4 contained either K267E or A291P (Figure 5).

### DPP4 polymorphisms K267E, K267N and A291P reduce MERS-CoV S binding efficiency to DPP4

**Figure 6.**
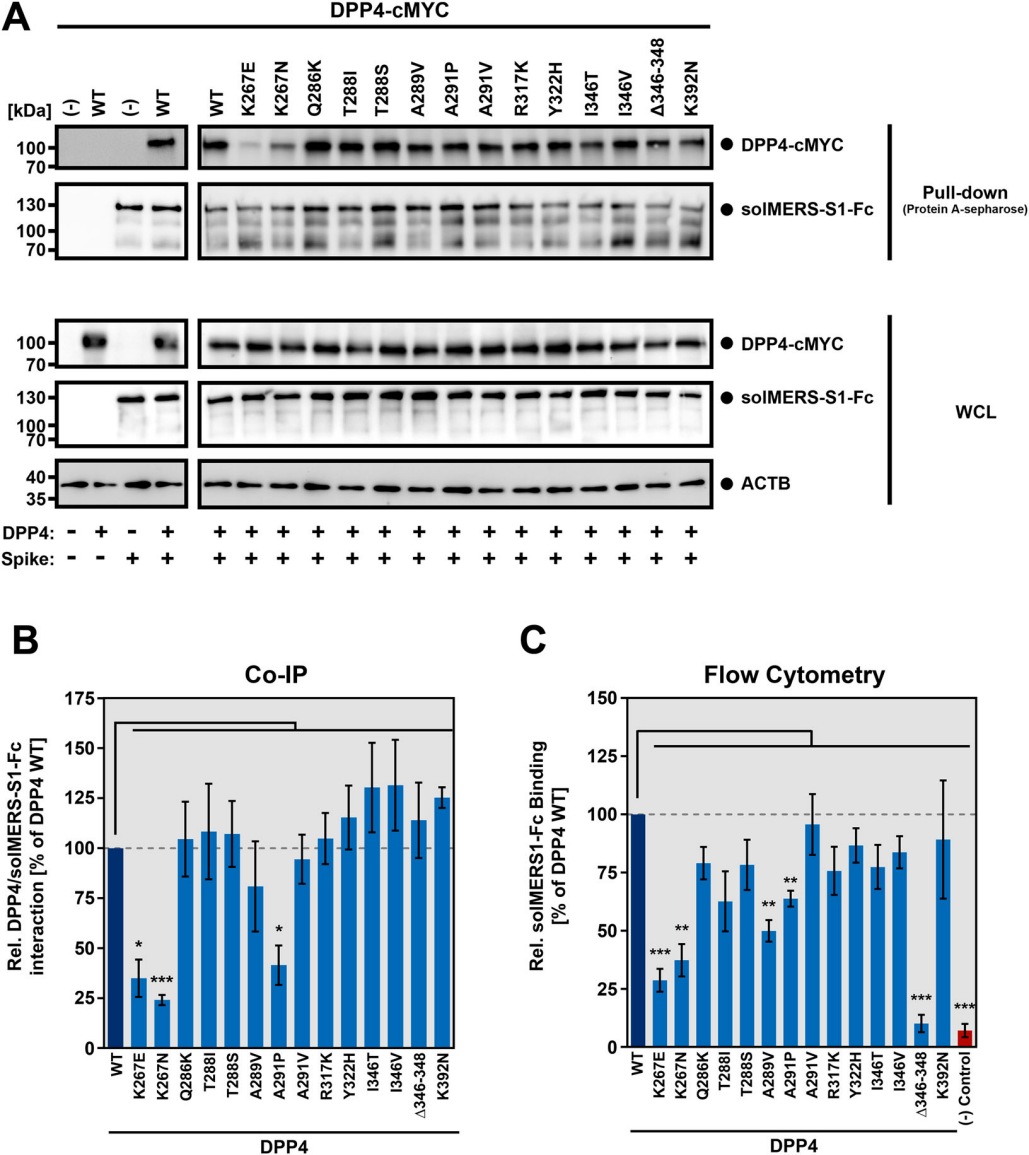
Reduced MERS-CoV S-driven host cell entry is caused by inefficient S protein binding to DPP4 harboring polymorphic amino acid residues. In order to investigate whether reduced MERS-CoV S-driven host cell entry and MERS-CoV replication is due to inefficient MERS-CoV S binding to DPP4 harboring amino acid polymorphisms at the binding interface, we performed co-immunoprecipitation (co-IP) as well as binding experiments with a soluble S protein comprising the S1 subunit of MERS-CoV S fused to the Fc region of human IgG. (A) 293T cells were cotransfected with expression plasmids coding for soluble, Fc-tagged MERS-CoV S1 (solMERS-S1-Fc) and the indicated DPP4 variant containing a C-terminal cMYC-tag. Cells that were transfected only with empty expression vector alone, or empty expression vector instead of either solMERS-S1-Fc or DPP4 served as controls. At 48 h posttransduction, cells were lysed and incubated with protein A sepharose. Next, samples were subjected to SDS-PAGE and Western blot analysis. DPP4 levels were detected via antibodies specific for the cMYC-tag, whereas solMERS-S1-Fc was detected using a peroxidase-coupled anti-human antibody. Similar results were obtained in three individual experiments. Analysis of whole cell lysates (WCL) for expression of solMERS-S1-Fc, DPP4 and ß-actin confirmed comparable ß-actin levels in each sample and comparable expression levels for solMERS-S1-Fc and DPP4. (B) For quantification of MERS-CoV S/DPP4 interaction we first normalized the DPP4 signals against the respective solMERS-S1-Fc signals. Then, MERS-CoV S/DPP4 interaction was set as 100% for wildtype (WT) DPP4 and the relative interaction efficiency for each DPP4 mutant was calculated accordingly. Presented are the mean data from three independent experiments. Error bars indicate the SEM. Statistical significance of differences in MERS-CoV S/DPP4 interaction between WT and mutant DPP4 was analyzed by one-way analysis of variance with Dunnett’s posttest (*p* > 0.05, ns; *p* ≤ 0.05, *; *p* ≤ 0.001, ***). (C) Soluble MERS-CoV S1-Fc was incubated with BHK-21 cells expressing wildtype (WT) or mutant DPP4, or cells transfected with empty expression vector or an ACE2-expression plasmid (controls). To detect bound S protein, the cells were subsequently incubated with an AlexaFluor488-conjugated anti-human antibody directed against the Fc-tag. Fluorescent signals representing bound solMERS-S1-Fc were analyzed by flow cytometry and MFI values for each sample were calculated. For normalization, the MFI value of the negative control (empty expression vector) was subtracted from all samples. Further, binding of solMERS-S1-Fc to cells expressing DPP4 WT was set as 100% and the relative binding to cells expressing the DPP4 mutants was calculated accordingly. Shown are the combined data of five independent experiments with error bars indicating the SEM. Statistical significance of differences in solMERS-S1-Fc binding to cells expressing WT or mutant DPP4 was analyzed by one-way analysis of variance with Dunnett’s posttest (*p* > 0.05, ns; *p* ≤ 0.05, *; *p* ≤ 0.01, **; *p* ≤ 0.001, ***).

After the identification of DPP4 polymorphisms that reduce S-driven cellular entry of rhabdoviral vectors as well as MERS-CoV replication, we next sought to investigate whether the attenuating phenotype was due to reduced binding of MERS-CoV S to DPP4. For this, we used soluble MERS-CoV S, produced by fusing the S1 subunit, which contains the DPP4 binding domain, to the Fc portion of human immunoglobulin G (solMERS-S1-Fc). Co-immunoprecipitation analysis demonstrated that DPP4 variants harboring polymorphisms K267E, K267N or A291P, which were not compatible with efficient MERS-CoV S-driven host cell entry, displayed significantly reduced ability to interact with MERS-CoV S as indicated by weaker DPP4 signals upon protein A-sepharose-mediated pull-down of DPP4/solMERS-S1-Fc (as compared to DPP4 WT, Figure 6A-B). Notably, DPP4 variant Δ346–348 could be as efficiently co-immunoprecipitated as DPP4 WT, indicating that its inefficient receptor function was solely due to its defect in proper surface transport. The findings obtained by co-IP analysis were confirmed by flow cytometry. It was revealed that polymorphisms that reduced MERS-CoV S-driven host cell entry (K267E, K267N, A291P and Δ346–348) and spread of authentic MERS-CoV (K267E and A291P) also reduced MERS-CoV S binding to cells expressing DPP4 on the cell surface (Figure 6C). In addition, polymorphism A289V, which decreased MERS-CoV S-driven transduction to a lesser extent than the aforementioned polymorphisms (Figure 4), also reduced MERS-CoV S binding to DPP4. Thus, DPP4 polymorphisms K267E, K267N and A291P reduce MERS-CoV S-driven host cell entry and MERS-CoV infection by diminishing MERS-CoV S binding to DPP4.

## Discussion

Host cell entry of MERS-CoV critically depends on the interaction between the viral S protein and the cellular receptor DPP4. A link between obesity or underlying diseases like diabetes mellitus, which both can affect DPP4 expression levels [48], and the risk of fatal outcome of MERS-CoV infection has been made [49]. Moreover, alanine scanning mutagenesis identified DPP4 residues critical for MERS-CoV entry, including K267, L294, I295, R317 and R336 [50,51]. However, the impact of natural-occurring variations on host cell entry of MERS-CoV has not been addressed so far. We identified DPP4 polymorphisms that reduce S protein-driven host cell entry and replication of authentic MERS-CoV by lowering the binding efficiency of MERS-CoV S to DPP4, suggesting that the DPP4 phenotype may impact the course of MERS-CoV infection.

Western blot analysis, flow cytometry and confocal laser scanning microscopy revealed that none of the polymorphisms studied, except deletion of amino acids 346–348, had a significant impact on total or cell surface expression of DPP4, at least in the context of DPP4 transfected cells. Four polymorphisms located at three different sites in DPP4 (K267E, K267N, A291P and Δ346–348) severely reduced S protein-driven host cell entry. As DPP4 Δ346–348 was shown to be incompatible with robust cell surface transport but able to interact with MERS-CoV S in co-IP analysis, we conclude that the reduction in entry efficiency is solely due to insufficient DPP4 surface levels. In contrast, reduction of host cell entry by K267E, K267N and A291P could not be explained by reduced DPP4 expression and these polymorphisms were thus further investigated. MERS-CoV infection of BHK-21 cells transfected to express DPP4 WT and variants K267E or A291P revealed that K267E or A291P were not compatible with robust MERS-CoV replication. Finally, co-IP analyses and binding studies with soluble MERS-CoV S showed that these DPP4 polymorphisms reduced S protein binding to DPP4.

When looking at the crystal structure of the complex consisting of the MERS-CoV S receptor binding domain bound to DPP4, these observations do not come as a surprise. DPP4 residue K267 has been reported to contact MERS-CoV S residues G538 and D539, including a salt bridge interaction with D539 [16]. The exchange of K267 to either glutamate (E) or asparagine (N) likely abolishes/decreases the interaction with MERS-S due to the different biochemical properties of K267 (positively charged, basic) versus E267 (negatively charged, acidic) and N267 (not charged, acidic) (Supplementary Figure 1). For DPP4 residue A291, which has been reported to contact the MERS-CoV S residue E513, no information on the type of interaction is available [16]. Here, we speculate that the bulky and distorted side chain of proline (in comparison to the small side chain of alanine) abolishes/decreases interaction with MERS-CoV S residue E513 (Supplementary Figure 1). In contrast to that, valine contains a small side chain and also has identical biochemical properties as alanine and thus might be efficiently contacted by E513 of MERS-CoV S, which is why we did not observe any impact of polymorphisms A291V on MERS-CoV S-driven entry and MERS-CoV S MERS-CoV S binding/interaction (Supplementary Figure 1).

The observation that certain polymorphisms in DPP4 reduced MERS-CoV S binding and viral entry triggered the question whether residues in MERS-CoV S that are in direct contact with the respective DPP4 residues are also polymorphic. Indeed, we obtained initial evidence to support such a concept. Thus, we found that residue 539 in MERS-CoV S which contacts DPP4 residue 267 is polymorphic, with certain MERS-CoV variants harboring an asparagine instead of an aspartate residue at this position. D539N reduced entry into cells expressing relatively low amounts of DPP4 but had no effect on entry into cells expressing high amounts of DPP4 (Supplementary Figure 2). Moreover, and more interestingly, D539N slightly rescued MERS-CoV S-driven entry from the negative effect exerted by DPP4 polymorphism K267N (Supplementary Figure 2). Similarly, residue 510 in MERS-CoV S, which is known to interact with DPP4 residues 317 and 322, was found to be polymorphic, and previous studies demonstrated that polymorphism D510G reduced DPP4 binding but also increased resistance to neutralizing antibodies [37]. Notably, D510G slightly increased entry via DPP4 harboring polymorphism Y322H and allowed MERS-CoV S to use DPP4 with polymorphism R317K with the same efficiency as WT DPP4. It should be stated that none of these effects was statistically significant and that DPP4 and MERS-CoV S polymorphisms occur with low frequency. Although it is unlikely that the DPP4 polymorphisms have emerged as a result of evolutionary pressure from MERS-CoV infections, our results suggest that certain existing DPP4 polymorphism(s) might foster the emergence of MERS-CoV variants with altered biological properties.

The polymorphisms studied here occur with relatively low frequencies of one per ∼19,000 (A289V) to ∼245,000 (T288I) individuals. However, detailed information on the geographic distribution or incidence in certain ethnical groups is largely missing. Thus, DPP4 polymorphisms could contribute to the perplexing absence of MERS cases in Africa, where the virus circulates in camels [52–57]. However, recent evidence suggests that sequence variations between African and Arabian MERS-CoV might be a factor [53,57]. More importantly, it remains to be analyzed how frequent DPP4 polymorphisms that affect S protein binding occur in the Middle East and whether they are associated with the clinical course of MERS.

## Supplementary Material

Supplemental Material
