# Supplementary material for: Polymorphisms in dipeptidyl peptidase 4 reduce host cell entry of Middle East respiratory syndrome coronavirus
Source: Emerg Microbes Infect. 2020 Jan 21;9(1):155–68. doi: 10.1080/22221751.2020.1713705 (PMC7006675; doi:10.1080/22221751.2020.1713705)
Supplement: Supplemental Material [file TEMI_A_1713705_SM6840.zip › Supplementary_table_1_final.docx]

**Supplementary Table 1**

**Table S1: Frequency of DPP4 polymorphisms according to the Ensembl database (<https://www.ensembl.org/>)**

| **Polymorphism** | **Database** | **n(total)^1^** | **n(poly)^2^** | **Frequency [%]** | **Population** |
| --- | --- | --- | --- | --- | --- |
| **K267E** | **all** | **125568** | **1** | **7,96381E-06** | **n/a** |
|  | gnomAD | n/a | n/a | n/a |  |
|  | TOPMed | 125568 | 1 | 7,96381E-06 |  |
|  | ExAC | n/a | n/a | n/a |  |
|  | 1000G | n/a | n/a | n/a |  |
| **K267N** | **all** | **365360** | **2** | **5,47405E-06** | **n/a** |
|  | gnomAD | 244362 | 1 | 4,09229E-06 |  |
|  | TOPMed | n/a | n/a | n/a |  |
|  | ExAC | 120998 | 1 | 8,2646E-06 |  |
|  | 1000G | n/a | n/a | n/a |  |
| **Q286K** | **all** | **30859** | **1** | **3,24055E-05** | **AFR** |
|  | gnomAD | 30859 | 1 | 3,24055E-05 |  |
|  | TOPMed | n/a | n/a | n/a |  |
|  | ExAC | n/a | n/a | n/a |  |
|  | 1000G | n/a | n/a | n/a |  |
| **T288I** | **all** | **245413** | **1** | **4,07476E-06** | **SAS** |
|  | gnomAD | 245413 | 1 | 4,07476E-06 |  |
|  | TOPMed | n/a | n/a | n/a |  |
|  | ExAC | n/a | n/a | n/a |  |
|  | 1000G | n/a | n/a | n/a |  |
| **T288S** | **all** | **366684** | **7** | **1,909E-05** | **SAS** |
|  | gnomAD | 245418 | 4 | 1,62987E-05 |  |
|  | TOPMed | n/a | n/a | n/a |  |
|  | ExAC | 121266 | 3 | 2,4739E-05 |  |
|  | 1000G | n/a | n/a | n/a |  |
| **A289V** | **all** | **492166** | **26** | **5,28277E-05** | **AMR, ASJ, EUR, OTH** |
|  | gnomAD | 245340 | 18 | 7,33676E-05 |  |
|  | TOPMed | 125568 | 4 | 3,18552E-05 |  |
|  | ExAC | 121258 | 4 | 3,29875E-05 |  |
|  | 1000G | n/a | n/a | n/a |  |
| **A291P** | **all** | **491948** | **13** | **2,64256E-05** | **EAS, EUR** |
|  | gnomAD | 245134 | 7 | 2,85558E-05 |  |
|  | TOPMed | 125568 | 3 | 2,38914E-05 |  |
|  | ExAC | 121246 | 3 | 2,47431E-05 |  |
|  | 1000G | n/a | n/a | n/a |  |
| **A291V** | **all** | **371388** | **3** | **8,07781E-06** | **EAS** |
|  | gnomAD | 245128 | 1 | 4,0795E-06 |  |
|  | TOPMed | n/a | n/a | n/a |  |
|  | ExAC | 121252 | 1 | 8,24729E-06 |  |
|  | 1000G | 5008 | 1 | 0,000199681 |  |
| **R317K** | **all** | **367312** | **2** | **5,44496E-06** | **EUR** |
|  | gnomAD | 245972 | 1 | 4,0655E-06 |  |
|  | TOPMed | n/a | n/a | n/a |  |
|  | ExAC | 121340 | 1 | 8,24131E-06 |  |
|  | 1000G | n/a | n/a | n/a |  |
| **Y322H** | **all** | **125568** | **2** | **1,59276E-05** | **n/a** |
|  | gnomAD | n/a | n/a | n/a |  |
|  | TOPMed | 125568 | 2 | 1,59276E-05 |  |
|  | ExAC | n/a | n/a | n/a |  |
|  | 1000G | n/a | n/a | n/a |  |
| **I346T** | **all** | **367580** | **4** | **1,0882E-05** | **AMR, EUR** |
|  | gnomAD | 246174 | 2 | 8,12433E-06 |  |
|  | TOPMed | n/a | n/a | n/a |  |
|  | ExAC | 121406 | 2 | 1,64737E-05 |  |
|  | 1000G | n/a | n/a | n/a |  |
| **I346V** | **all** | **246972** | **3** | **1,21471E-05** | **n/a** |
|  | gnomAD | n/a | n/a | n/a |  |
|  | TOPMed | 125568 | 2 | 1,59276E-05 |  |
|  | ExAC | 121404 | 1 | 8,23696E-06 |  |
|  | 1000G | n/a | n/a | n/a |  |
| **Δ346-348** | **all** | **277133** | **2** | **7,21675E-06** | **AFR** |
|  | gnomAD | 246180 | 1 | 4,06207E-06 |  |
|  | TOPMed | 30953 | 1 | 3,2307E-05 |  |
|  | ExAC | n/a | n/a | n/a |  |
|  | 1000G | n/a | n/a | n/a |  |
| **K392N** | **all** | **246504** | **2** | **8,11346E-06** | **EUR** |
|  | gnomAD | 131500 | 1 | 7,60456E-06 |  |
|  | TOPMed | 115004 | 1 | 8,69535E-06 |  |
|  | ExAC | n/a | n/a | n/a |  |
|  | 1000G | n/a | n/a | n/a |  |

^1^: Number of samples/patients analyzed, ^2^: Number of samples/patients for which a specific polymorphism was found

Abbreviations: AFR = African/African American, AMR = Latino, ASJ = Ashkenazi Jewish, EAS = East Asian, EUR = European; SAS = South Asian, OTH = others, n/a = not available

Sources: gnomAD (<https://gnomad.broadinstitute.org/>), TOPMed (<https://www.nhlbiwgs.org/>), ExAC (<http://exac.broadinstitute.org/>) and 1000G (<http://www.internationalgenome.org/>).
