# Supplementary figures and images for: Polymorphisms in dipeptidyl peptidase 4 reduce host cell entry of Middle East respiratory syndrome coronavirus
Source: Emerg Microbes Infect. 2020 Jan 21;9(1):155–68. doi: 10.1080/22221751.2020.1713705 (PMC7006675; doi:10.1080/22221751.2020.1713705)

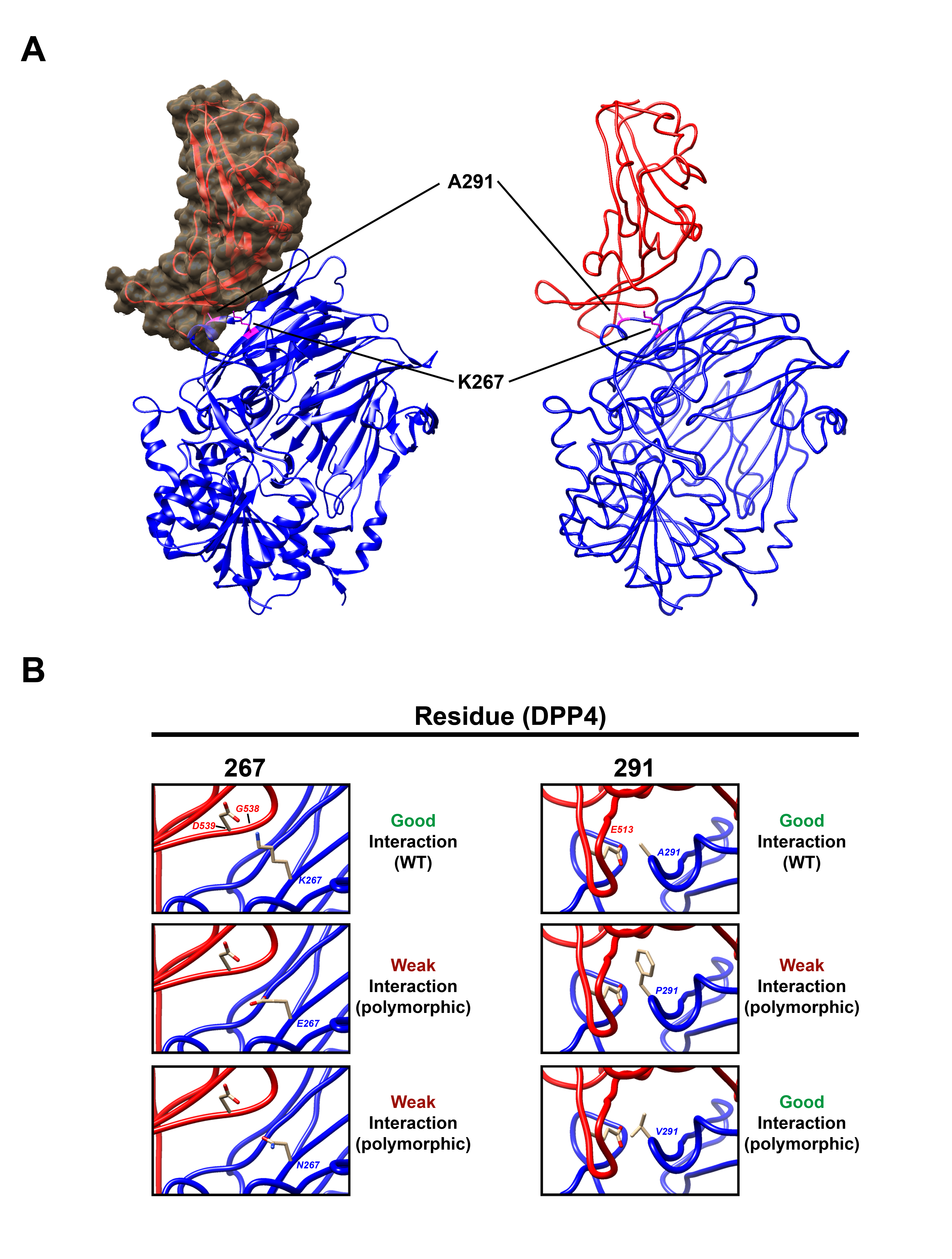

Supplement: Supplemental Material [file TEMI_A_1713705_SM6840.zip › Supplementary_figure_1_final.tif]

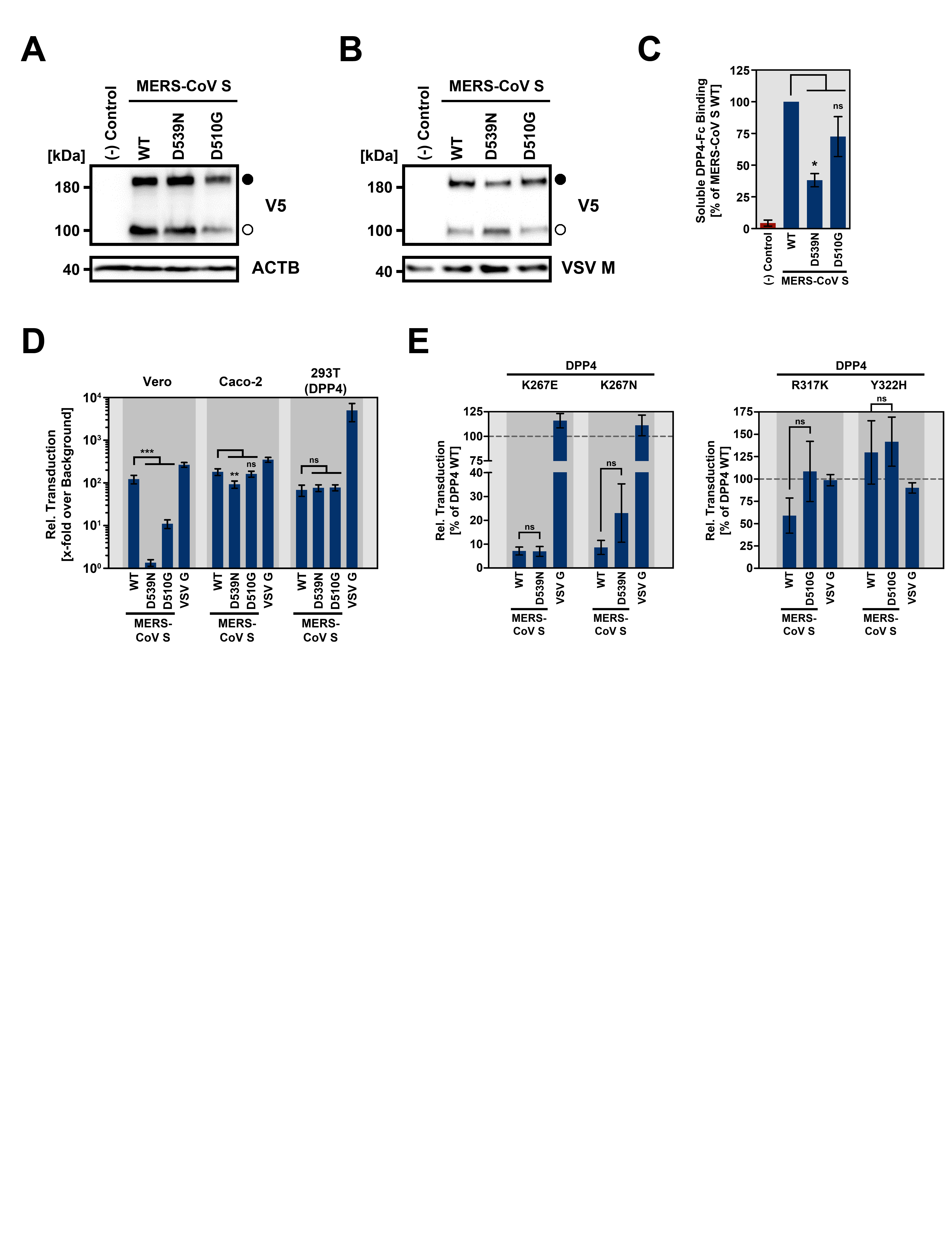

Supplement: Supplemental Material [file TEMI_A_1713705_SM6840.zip › Supplementary_figure_2_final.tif]
